# Dataset of the impact of food insecurity on health outcomes in sub-Saharan Africa

**DOI:** 10.1186/s13104-023-06623-5

**Published:** 2023-11-25

**Authors:** Sisay Demissew Beyene

**Affiliations:** Department of Economics, Arsi University, Asella, Ethiopia

**Keywords:** Food insecurity, Health outcomes, Secondary panel data, Yearly balanced data, International institutions database, SSA countries, STATA 15

## Abstract

**Objectives:**

The 2030 Sustainable Development Goals (SDGs) have goals and targets, including food insecurity and health outcomes. Hence, information about socioeconomic variables that determine the health outcomes of people is essential for health-related research, planning, and policy development. Therefore, this data paper aims to present (describe) the dataset of eight socioeconomic variables for 31 sub-Saharan Africa (SSA) countries from 2001 to 2018.

**Data descriptions:**

The dataset was official information obtained via open online sources from the Food and Agricultural Organization (FAO), the World Bank (WB), and the United Nations Development Programme (UNDP). It included 558 observations and eight variables, such as life expectancy (LEXP), infant mortality (INFMOR), the prevalence of undernourishment (PRUND), average dietary energy supply (AVRDES), Gross domestic product per capita (GDPPC), general government health expenditure (GOVEXP), urbanisation (URBAN), and mean years of schooling (MNSCHOOL). Moreover, all the data estimation is conducted by Statistical Software (STATA) version 15. This paper achieved its intended objective with a detailed and understandable description.

**Supplementary Information:**

The online version contains supplementary material available at 10.1186/s13104-023-06623-5.

## Objectives

Several factors determine human health outcomes. Specifically, food insecurity and health outcomes are inseparable and are the agenda of the 2030 SDGs, researchers, and policymakers. Thus, awareness of the socioeconomic factors that affect people’s health outcomes is crucial to provide evidence-based findings and developing achievable and comprehensive policies. Therefore, this data paper aims to describe a dataset used in Beyene’s [1] article, which is freely and openly accessed at 10.6084/m9.figshare.22606442.v1 [2]. Specifically, this paper seeks to provide a detailed overview of the data sources, data collection methodology, and data quality. By doing so, researchers can better understand the context in which the data was gathered, its accuracy and its implications for the research.

There is evidence for the rationale for the research and collecting the data in relation to food insecurity and health in the context of SSA. According to Roser and Ritchie [3], food insecurity is highest in SSA nations, where approximately one-third of the population is classified as highly insecure. Besides, 11% (820 million) of the world’s population was malnourished in 2018, with SSA nations continuing to bear a more significant burden [3]. Likewise, Word Count [4] reported that the amount of people suffering from lack of food has grown since 2015; hence, meeting the SDGs of ending hunger by 2030 would be a major problem [4]. Since food insecurity is one of the determinantal factors for health outcomes, the author was motivated to collect the data and examine the implication of food insecurity on life expectancy and infant mortality in SSA countries.

## Data descriptions

The dataset is secondary panel macro-level yearly data collected (via open online databases) from well-known institutions, such as FAO [5], the WB [6], and the UNDP [7]. The dataset has 558 observations and eight socioeconomic variables (for more details, see Data File 1 in Table 1). These variables are LEXP,^1^ INFMOR,^2^ PRUND,^3^ AVRDES,^4^ GDPPC, GOVEXP (% GDP), URBAN,^5^ and MNSCHOOL. Except for the mean years of schooling and average dietary energy supply, all variables were collected from WB. However, the mean years of schooling were obtained from UNDP, and the average dietary energy supply data were FAO data obtained from Knoema. The LEXP and INFMOR, dependent variables, are proxies of health outcomes and are transformed into natural logarithms.^6^ On the other hand, PRUND and AVRDES are proxy variables for food insecurity and are the target independent variables. All independent variables, except for GDPPC and MNSCHOOL, were measured in percentage. However, GDPPC is measured in constant 2010$ while MNSCHOOL is in the number of years (for more details, see Table 2 of a summary).

**Table 1:** Overview of data files/datasets

Lebel	Name of datafile/dataset	File type/ file extension	Data repository and identifier (DOI number)
Data file 1	Additional file 3	Excel (.xlxs)	10.6084/m9.figshare.22606442.v1 [2]
Data file 2	Additional file 2	.docx	10.6084/m9.figshare.22606439.v1 [8]

**Table 2:** Summary of the variables sources

Variables, definitions, and measurements	Source links
LNLEXP is the natural logarithm of life expectance at birth measured in years	https://databank.worldbank.org/source/world-development-indicators#
LNINFMOR is the natural logarithm of infant mortality rate per 1000 lives birth before 1 year old	
PRUDN refers to the prevalence of undernourishment, indicating the % of the population whose habitual food consumption is insufficient to supply the dietary energy levels essential to living a normally active and healthy life	
GDPPC is GDP per capita measured based on the 2010 US$ constant price	
GOVEXP stands for domestic general government health expenditure (% of GDP)	
URBAN refers to urbanization measured as urban population (% of the total population)	
MNSCHOOL is mean years of schooling	http://hdr.undp.org/en/data
AVRDES is the average dietary energy supply adequacy measured as % (3-year average)	https://knoema.com/FAOFSD2020/fao-food-security-data?location=1000180-sub-saharan-africa

The data were collected in several steps. First, the study selected the target and the control variables along with their proxies based on the existing literature. Second, to have long time-series data and a large sample size, the data was started in 1990 and included for all SSA countries. Third, the author searched for data from all available online sources; however, due to data availability for most variables and countries as well as for the sake of consistency, the data were collected only from the WB, UNDP, and FAO. However, the data for target variables, such as the prevalence of undernourishment and average dietary energy supply not available before 2001 in most SSA countries. Moreover, some countries have no data on the prevalence of undernourishment, even since 2001. Hence, since the balanced data is better than the unbalanced one, the author has excluded some countries with no or missing data and finally arrived at 31^7^ sampled SSA countries from 2001 to 2018. The collected data were recorded in an Excel sheet and imported to STATA 15 for analysis. Further, before the final estimation, the most efficient model was selected (for more details, see Additional file 1).

## Limitations

Since the data collection was conducted at the end of 2020, the data ended in 2018. Thus, the data did not consider the recent global events, such as the Coronavirus disease of 2019 and the Russia-Ukrain war. In addition, the data were estimated using STATA 15. Moreover, the data was only focused on socioeconomic factors of health outcomes. Therefore, other scholars can extend the year until 2022, consider the environmental and political factors, and employ the latest statistical software (STATA 17).

### Supplementary Information

**Additional file 1:** Additional documentation and Algorithms.

## Data Availability

The data described in this data note is freely and openly available at 10.6084/m9.figshare.22606442.v1 [2] and in Table 1.

## Abbreviations

AVRDES: Average dietary energy supply
FAO: Food and Agricultural Organization
GDPPC: Gross domestic product per capita
GOVEXP: General government health expenditure
INFMOR: Infant mortality
LEXP: Life expectancy
MNSCHOOL: Mean years of schooling
PRUND: Prevalence of undernourishment
SDGs: Sustainable Development Goals
SSA: Sub-Saharan Africa
STATA: Statistical Software
UNDP: United Nations Development Programme
URBAN: Urbanisation
WB: World Bank

## Footnotes

1 Transformed to natural logarithm and measured in the number of years.

2 Transformed to natural logarithm and indicates infants dying before one year old (per 1000 live births).

3 The percentage of people whose regular food intake is inadequate to meet the dietary energy levels needed to. maintain an active and healthy lifestyle.

4 Measured as dietary energy supply as a proportion of average dietary energy needs (3-year average). The average. supply of calories for food consumption in each nation or area is adjusted by the mean dietary energy demand estimated for its people to create an index of food supply adequacy in calorie terms.

5 Measured as the urban population as a percentage of the total population.

6 Important to measure the elasticity and to reduce the gaps between the raw data’s variable values.

7 Angola, Botswana, Benin, Burkina Faso, Cabo Verde, Cameroon, Congo Rep., Chad, Cote d’Ivoire, Gabon, Ethiopia, The Gambia, Kenya, Ghana, Liberia, Lesotho, Malawi, Madagascar, Mauritania, Mali, Mozambique, Mauritius, Nigeria, Namibia, Senegal, Rwanda, South Africa, Sierra Leone, Tanzania, Sudan, and Togo.
