# Supplementary material for: Dataset of the impact of food insecurity on health outcomes in sub-Saharan Africa
Source: BMC Res Notes. 2023 Nov 25;16:349. doi: 10.1186/s13104-023-06623-5 (PMC10675889; doi:10.1186/s13104-023-06623-5)
Supplement: Supplementary file 1 — Additional file 1: Additional documentation and Algorithms. [file 13104_2023_6623_MOESM1_ESM.docx]

**Justifications for the use of the WB, UNDP, and FAO data**

The summary table (Table 2) shows that the WB, UNDP, and FAO are the three main sources of data due to their trustworthy data and well-known international institutions. According to the Guardian [1], WB and UNDP are among the top 10 sources of data for international development research. Studies confirmed that UNDP ranks among the top four information providers in low and middle-income countries [2].

In the Table 2, seven out of nine (78%) variables were collected from WB since the WB applies internationally accepted standards and norms, resulting in a consistent, reliable source of information. The WB adheres to professional standards for data collection, compilation, and dissemination, ensuring data quality and integrity [3].

Statistics must be reliable, relevant, and meet the needs of users and policymakers. In this regard, developing countries, including SSA, often face challenges. To overcome this, the WB is working to improve the quality of statistics in developing countries by investing in statistical activities, creating standards for data collection and dissemination, strengthening the international statistical system, and compiling global data sets [3]. Hence, since the case study of the paper is SSA, using the WB data is more reliable. Since the WB collaborates with international statistical communities like the UN, OECD, IMF, regional development banks, and donors, and establishes data exchange and dissemination processes, we can get the collaborators’ data compiled by the WB easily. The WB provides the data via an Open Data site, which is easy to find, download, and use free of charge [3].

The WB data has several data, such as the World Bank Data Portal, World Development Indicators, World Development Report, International Debt Statistics, Global Consumption Database, Subnational Population Database, and Database of Political Institutions [4]. Among these, the author employed the WDI since it has a massive collection of national data on hundreds of indicators [1] with six dimensions: World View, People, Environment, Economy, States/Markets, and Global Links [4]. It goes back a long way (since 1960) and has data on every country in the world, including SSA [1, 4].

Similarly, the UNDP produces an enormous amount of data, insight, and analysis [2]. It also provides trustful data by implementing principles that promote professional independence, impartiality, scientific and transparent methods, and equal access to official statistical information [5]. Likewise, the FAO is dedicated to collecting, analyzing, interpreting, and disseminating relevant statistics for decision-making in food and agriculture issues. It develops methodologies and standards to assist countries in generating sound data and information [6]. Specifically, FAOSTAT provides free access to food and agriculture data for over 245 countries and territories and covers all FAO regional groupings from 1961 to the most recent year available [6]. It provides high-quality data since it has a Statistics and Data Quality Assurance Framework (SDQAF) and Corporate Statistical Standards [6]. FAO also collaborates with the Regional Commission for Agriculture Statistics in Africa (AFCAS), which is one of the statutory bodies of FAO on statistics [6].

**Detailed Methodology with STATA commands**

The variables included in the models selected based on the existing literature. Since the variables are macro-level, available for most SSA countries, as well as for the sake of consistency, the data were collected only from the WB, UNDP, and FAO. Although the methods of data collecting provided by each institution differ, these sources give data online at no cost. In more detail, the author used the [World Bank open data](https://data.worldbank.org/about) and downloaded the bulk Excel file version of the [World Development Indicators database](https://datatopics.worldbank.org/world-development-indicators/). Similarly, I have used the [UNDP’s download and documentation](https://hdr.undp.org/data-center/human-development-index#/indicies/HDI) section and downloaded the data link entitled “[All composite indices and components time series Metadata](https://hdr.undp.org/data-center/documentation-and-downloads)”. After I had signed up for the [Knoema data source](https://knoema.com/sys/login/signup?returnUrl=%2FFAOFSD2020%2Ffao-food-security-data%3Flocation%3D1000180-sub-saharan-africa), which has the FAO data about AVRDES, I downloaded the Excel version of the dataset. After downloading all datasets from the three sources, based on the model variables, I selected the required data. The author initially included all SSA countries since 1990 in the sample but found data for target variables like undernourishment prevalence and average dietary energy supply unavailable before 2001. Some countries also had no data on undernourishment prevalence since 2001. Hence, since the balanced data is better than the unbalanced, the author has excluded some countries with no or missing data and finally arrived at 31 sampled SSA countries from 2001–2018.

Panel data analysis utilizes various estimation techniques, primarily based on basic panel econometric tests like CD, unit root, and cointegration tests. CD is crucial in dynamic panel estimators, and overlooking it can result in biased estimates and spurious outcomes. Hence, the author employed Pesaran [7], Frees [8], and Friedman [9] CD tests.

Note: Since Beyene’s study has four basic and other alternative models with several variables, for the sake of understanding and to save space, DV in this document represents the dependent variable, while INDPV/s refers to the independent variable/s. Further, all texts in the quotations are Stata commands.

Before the basic panel econometric tests, the author exported the collected Excel data to Stata version 15 using copy and paste and coded (given ID for the sampled countries) using ***“encode country, gen(id)”*** and introduced the software; the type of data is panel by ***“tsset id year, yearly”***. After the regression ***“xtreg DV INDPVs”***, the CD tests were conducted in three ways: (1) Pesaran – ***“xtcsd, pesaran abs”*** (2) Frees – ***“xtcsd, frees”***, (3) Friedman – ***“xtcsd, friedman”***.

The CD test determines the type of unit root and cointegration tests to apply, which are common steps after the CD test. There are two types of unit root tests: first-generation and second-generation. First-generation tests assume cross-sectional independence, which is restrictive due to macroeconomic time series correlations. Second-generation tests consider cross-sectional correlation and can generate size distortions. Hence, the author used Pesaran’s [10] cross-sectionally augmented panel unit root test (CIPS) for each variable in **models 1A–1C**. The level unit root test command for each variable (say LNLEXP) is ***“xtcips lnlexp, maxlags(2) bglags(1) q”*** while the first difference is ***“xtcips d.lnlexp, maxlags(2) bglags(1) q”***.

However, since there is no CD in **model 1D**, the author employed the first-generation unit root tests called Levin, Lin, and Chu (LLC), Im, Pesaran, Shin (IPS), and Fisher augmented Dickey-Fuller (ADF) for **model 1D**. Thus, the level LLC command for each variable (say GDPPC) is ***“xtunitroot llc gdppc, lag(0)”*** while the first difference is ***“xtunitroot llc d.GDPPC, lag(0)”***. Besides, the level IPS command is ***“xtunitroot ips GDPPC, demean”*** while the first difference is ***“xtunitroot ips d.GDPPC, demean”***. The Fisher command is ***“xtunitroot fisher GDPPC, dfuller lags(0)”*** for level tests and ***“xtunitroot fisher d.GDPPC, dfuller lags(0)”*** at the first difference.

Among the existing panel cointegration tests that allow CD, I used Westerlund [11] and Banerjee and Carrion-i- Silvestre [12] for **models 1A–1C**. The command for only target variables is ***“xtwest DV INDPV, constant trend lags(1) leads(1) lrwindow(3) bootstrap(100)”***. However, the Carrion-i- Silvestre [12] steps for all variables are: (1) ***“xtdcce2 d.DV l.DV INDPVs, reportc cr(DV INDPVs) pooled(L.DV INDPVs) noconstant”***, (2) ***“predict residuals” and (3) “xtcips residuals, maxlags(2) bglags(1) q”***.

However, to decide uncertain results, the McCoskey and Kao [13] cointegration tests was used for **model 1C**. For models with target variables, the steps are: (1) ***“xtcointreg DV INDPV, est(dols) noconstant”***, (2) ***“predict residuals”***, and (3) ***“xtunitroot ips residuals”***. For all variables, the commands are: (1) ***“xtcointreg DV INDPVs, est(dols) noconstant”***, (2) ***“predict residuals”, and (3) “xtunitroot ips residuals***”

The author utilized the Pedroni [14,15] cointegration test for **model 1D**, which is applicable when there is no CD and considers heterogeneity using ***“xtcointtest pedroni DV INDPVs, ar(same)”*** command.

The panel data analysis can be conducted using different estimation techniques and is mainly determined by the results of basic panel econometric tests. Thus, the author mainly employs the Driscoll-Kraay [16] standard error (DKSE) (for **models 1A and 1B**), FE (for **model 1C**), and two-step GMM (for **model 1D**) estimation techniques to examine the impact of food insecurity on health outcomes. However, the DKSE regression can be estimated in three ways: FE with DKSE, RE with DKSE, and pooled Ordinary Least Squares/ Weighted Least Squares (pooled OLS/WLS) regression with DKSE. Hence, we must choose the most efficient model using Hausman and Breusch-Pagan LM for RE tests.

The author employed various estimation techniques, including Driscoll-Kraay standard error (DKSE) for **models 1A and 1B**, FE for **model 1C**, and two-step GMM for **model 1D**. The DKSE regression can be estimated in three ways: FE with DKSE, RE with DKSE, and pooled OLS/WLS regression with DKSE. The most efficient model for RE tests is chosen using Hausman and Breusch-Pagan LM. The steps for the Hausman test are: (1) ***“xtreg DV INDPVs, fe”,*** (2) ***“estimates store fe”,*** (3) ***“xtreg DV INDPVs, re”,*** (4) ***“estimates store re”,*** (5) ***“hausman fe re, sigmamore”,*** and (6) ***Decision: If the prob. value <0.05, FE is more efficient than RE model***. The step for the Breusch-Pagan LM test: (1) ***“xtreg DV INDPVs, re”,*** (2) ***“xttest0”,*** (3) ***Decision: If the prob. value<0.05, RE is more efficient than pooled OLS***. Then, I employed FE with DKSE for **models 1A and 1B** using ***“xtscc DV INDPVs, fe lag(4)”*** and ***“xtreg DV INDPVs, fe”*** for **model 1C**. Moreover, the two-step GMM commands for **model 1D** are: (1) ***“xtabond2 DV l.DV INDPVs, gmm(l.DV, collapse) iv(INDPVs) noleveleq twostep orthogonal small”,*** (2) ***“nlcom (_b[l.lninfmor])/ (1-_b[L1.lninfmor])”,*** (3) ***“nlcom (_b[avrdes])/ (1-_b[L1.lninfmor])”,*** (4) ***“nlcom (_b[govexp])/ (1-_b[L1.lninfmor])”.*** For the robustness check, the author employed FMOLS, FGLS, and PCSE estimation techniques for *models 1A and 1B*. The command for FMOLS is ***“xtcointreg DV INDPVs, est(fmols) noconstant”*** while for FGLS is ***“xtgls DV INDPVs, panels(correlated)”***. The following command also used ***“xtpcse DV INDPVs”*** to estimate PCSE.

Furthermore, even though the Hausman test confirms that the FE is more efficient, I have employed the RE for **model 1C** using ***“xtreg DV INDPVs, re”.*** In addition, among the existing panel ARDL models (PMG, MG, and DFE), the author employed DFE for **model 1D***, which is* selected based on the Hausman test using the following commands: (1) ***“xtpmg d.DV d.INDPV d.INDPV, lr(l.DV INDPVs) ec (ECT) replace pmg”,*** (2) ***“estimates store pmg”,*** (3) ***“xtpmg d.DV d.INDPV d.INDPV, lr(l.DV INDPVs) ec (ECT) replace mg”,*** (4) ***“estimates store mg”,*** (5) ***“xtpmg d.DV d.INDPV d.INDPV, lr( l.DV INDPVs) ec (ECT) replace dfe”,*** (6) ***“estimates store dfe”,*** (7) ***hausman mg pmg, sigmamore,*** (8) ***Decision: If the prob. value>0.05, pmg is efficient than mg model,*** (9) ***“hausman mg dfe, sigmamore”,*** (10) ***Decision: If the prob. value>0.05, dfe is efficient than mg model,*** (11) ***“hausman pmg dfe, sigmamore”,*** (12) ***Decision: If the prob. value>0.05, dfe is efficient than pmg model.***

The author also conducted further analysis, such as descriptive statistics, correlation analysis, and initial diagnosis. The correlation analysis was conducted by ***“cor DV INDPVs”*** while the descriptive statistics of each variable were examined using ***“summarize each variable, detail”.*** The initial diagnosis, like cross‑sectional and time‑specific fixed effects is analyzed as follows: (1) ***“rename id c_id”,*** (2) ***“reg DV INDPVs c_id, robust”,*** (3) ***“testparm c_id”,*** (4) ***“reg DV INDPVs i.c_id i.year”,*** (5) ***“testparm i.c_id i.year”,*** (6) ***“reg DV INDPVs i.c_id i.year, robust”,*** (7) ***“testparm i.c_id i.year”***

The normality, heteroscedasticity, multicollinearity, and serial correlation tests were conducted. A command for the normality test is***“xtreg DV INDPVs”*** and then ***“xtsktest, reps(500)”***. For the heteroscedasticity test, the author employed ***“xttest3”*** after regression ***“xtreg DV INDPVs, fe”***. The multicollinearity test was conducted using ***“estat vif”*** after ***“regress DV INDPVs”.*** A command ***“xtserial DV INDPVs, output”*** is used for the serial correlation test. Finally, the outliers were detected using Cook D using: (1) ***“reg DV INDPVs”,*** (2) ***“predict D, cooksd”,*** and (3) ***“clist country DV INDPVs D if D> 4/558, noobs”.***

**References**

The Guardian. The top 10 sources of data for international development research. 2016. <https://www.theguardian.com/global-development-professionals-network/2016/mar/16/the-top-10-sources-of-data-for-international-development-research>. Accessed 07 Nov 2023.

Dieye AM. UNDP as ‘knowledge frontier’. 2018. <https://www.undp.org/blog/undp-knowledge-frontier>. Accessed 05 Nov 2023.

World Bank. About us. <https://data.worldbank.org/about> (2023). Accessed 08 Nov 2023.

1. European University Institute. The World Bank Data. <https://www.eui.eu/Research/Library/ResearchGuides/Economics/Statistics/DataPortal/WorldBankData> (2023). Accessed 06 Nov 2023.

UNDP. Principles governing international statistical activities. <https://hdr.undp.org/data-center/documentation-and-downloads> (2023). Accessed 08 Nov 2023.

1. FAO. Statistics. <https://www.fao.org/statistics/en/> (2023). Accessed 07 Nov 2023.
2. Pesaran, MH. General diagnostic tests for cross-section dependence in panels. 2004. IZA Discussion Paper No. 1240, University of Cambridge.
3. Frees, EW. Assessing cross-sectional correlation in panel data. Journal of Econometrics. 1995; 69(2): 393-414.
4. Friedman, M. The use of ranks to avoid the assumption of normality implicit in the analysis of variance. Journal of the American Statistical Association. 1937; 32(200): 675-701.
5. Pesaran, MH. A simple panel unit root test in the presence of cross‐section dependence. Journal of Applied Econometrics. 2007; 22(2): 265-312.
6. Westerlund J. Testing for error correction in panel data. Oxford Bulletin of Economics and Statistics. 2007; 69(6):709-48.
7. Banerjee A., Carrion‐i‐Silvestre, JL. Testing for panel cointegration using common correlated effects estimators. Journal of Time Series Analysis. 2017; 38(4): 610-36.
8. McCoskey S., Kao CA. Residual-based test of the null of cointegration in panel data. Econometric reviews. 1998; 17(1): 57-84.
9. Pedroni, P. Critical values for cointegration tests in heterogeneous panels with multiple regressors. Oxford Bulletin of Economics and Statistics. 1999; 61(S1): 653-70.
10. Pedroni, P. Panel cointegration: asymptotic and finite sample properties of pooled time series tests with an application to the PPP hypothesis. Econometric theory. 2004; 20(3): 597-625.
11. Driscoll, JC., Kraay, AC. Consistent covariance matrix estimation with spatially dependent panel data. Review of Economics and Statistics. 1998; 80(4): 549-60.
